# Children's media engagement and media literacy in platformized environments: primary school teachers' perspectives

**DOI:** 10.3389/fpsyg.2026.1847155

**Published:** 2026-06-22

**Authors:** Şükran Calp, Pınar Bulut

**Affiliations:** 1 Department of Primary Education, Faculty of Education, Düzce University, Düzce, Türkiye; 2 Department of Primary Education, Faculty of Education, Gazi University, Ankara, Türkiye

**Keywords:** digital platforms, media literacy, platform imperialism, platformization of childhood, primary school teacher

## Abstract

**Introduction:**

This study explores primary school teachers' interpretations of children's media engagement, the ways media content is reflected in children's values and behaviors, and the pedagogical positioning of media literacy within platform-dominated digital environments. Grounded in debates on platform imperialism, the study examines how teachers make sense of children's everyday encounters with global digital platforms and how they position media literacy in response to these conditions. In doing so, it addresses four interrelated questions concerning students' media engagement, value and behavioral reconfigurations, the role of global platforms, and teachers' pedagogical responses.

**Methods:**

A qualitative case study design was employed. Data were collected through semi-structured interviews with 71 primary school teachers working in public schools across different regions of Türkiye, including village, town, and city settings. Participants represented diverse age groups and levels of professional experience. The interview data were analyzed through thematic analysis. Both inductive and deductive procedures were used in order to identify recurrent patterns of meaning across teachers' accounts, while concepts such as platform imperialism, cultural autonomy, and pedagogical resistance provided an analytical frame for interpretation.

**Results:**

According to teachers' accounts, children's media experiences were increasingly organized within algorithmically structured and platform-centered environments. Teachers described these environments as reshaping authority relations, value orientations, and everyday practices. They associated children's media engagement with patterns such as platform concentration, normalization of risk, fragile critical filtering, moral ambiguity, consumption-oriented value formation, and shifts in epistemic authority from institutional actors toward digital platforms. Teachers also described family mediation as a site of tension, sometimes reinforcing and at other times partially limiting platform influence. In response to these perceived conditions, teachers positioned media literacy not simply as a technical competence but as a pedagogical orientation involving critical verification, ethical awareness, resistance to manipulation, and participatory engagement.

**Discussion:**

The findings suggest that children's media engagement is interpreted by teachers not merely as an issue of individual media use, but as part of a broader restructuring of childhood within platform-dominated environments. In this context, media literacy emerged in teachers' narratives as a contextually grounded pedagogical response to algorithmic authority, information fragility, and shifting cultural norms. The study contributes to discussions of childhood, digital culture, and education by offering an empirically situated account of how platformized environments are understood and negotiated in primary school contexts.

## 1 Introduction

Media literacy, platform imperialism, and childhood constitute interrelated analytical domains for examining children's media experiences in the context of global digital platforms. Children's engagement with media can be understood as taking place within broader cultural, economic, and ideological conditions shaped by platform-based systems, rather than solely as a matter of individual technology use. Integrating insights from critical media literacy, platform studies, and childhood research allows for an analytical framework in which media is approached as a pedagogical space, a site of cultural negotiation, and a context in which power relations become visible. Within this framework, media literacy serves as a key concept for exploring pedagogical resistance, platform dominance, and questions of cultural autonomy.

## 2 Theoretical framework

### 2.1 Media literacy and pedagogical resistance

Media literacy has long been defined as an individual cognitive skill domain or a set of competencies. Traditionally, media literacy has been conceptualized as a process or set of skills grounded in critical thinking, and the primary aim of media literacy education has been described as revealing the underlying implicit structures in media messages in order to cultivate aware audiences, critical observers, and informed citizens (Bulger and Davison, 2018). However, in recent years, there has been a growing need for a more multidimensional and contemporary perspective on media literacy. In this regard, contemporary critical pedagogy approaches propose rethinking this competence as a tool for cultural autonomy and social resistance (Kellner and Share, 2007). As emphasized by Giroux (2020), media culture functions not merely as a channel for transmitting information but as a form of “cultural pedagogy” that actively constructs ideological frameworks. According to Giroux, media operates as an educational sphere that reproduces social values, identities, and power relations; therefore, media literacy should extend beyond students' ability to decode media messages and enable them to recognize how these messages are connected to broader structures of social power (Giroux, 2014).

In alignment with Paulo Freire's (1970) concept of *critical consciousness*, media literacy pedagogy holds the potential to transform individuals from passive consumers into active producers of meaning, a process closely linked to pedagogical resistance. Within this framework, learning occurs as students develop an inquisitive stance through active engagement with media texts. Kellner and Share (2019) emphasize that such a pedagogy should not only focus on the content of media messages but also make visible the conditions of media production, ownership relations, and ideological influences. In this context, the role of teachers is not limited to encouraging children to interpret media content; rather, it involves supporting the development of ethical, cultural, and social awareness in relation to media. Buckingham (2003) argues that media education is intrinsically connected to a democratic understanding of citizenship and that its primary aim should be to teach children not *what* to think, but *how* to question. Accordingly, media literacy can be conceptualized as a critical learning space in which pedagogical resistance and cultural autonomy are actively constructed.

### 2.2 Platform imperialism and children's media

Media literacy, defined as a critical learning domain, has become increasingly significant for individuals confronted with global platforms. Today, global digital platforms function not only as channels for accessing information but also as powerful mediators of cultural production and identity formation. In what Van Dijck et al. (2018) describe as the “platform society”—a globalized online platform ecosystem in which social and economic flows are driven by algorithms and fueled by data—digital platforms penetrate the core of societies, reshape markets, transform social and civic practices, and influence democratic processes. The forces that construct the platform society pursue policies that are both capitalist and imperialist in nature.

As conceptualized by Jin (2015), “platform imperialism” refers to the reproduction of unequal power relations in the digital economy at the cultural and ideological levels. Platforms such as YouTube, TikTok, and Instagram function as central hubs through which global capital increasingly shapes content flows, thereby reshaping representations of local cultures and children's media experiences. This transformation is particularly influential during childhood, when values and behavioral patterns internalized through media play a decisive role in development. Livingstone and Blum-Ross (2020) emphasize that digital platforms redefine children's modes of learning and senses of cultural belonging. Similarly, Buckingham (2019b) argues that children's media is now produced in close alignment with the economic logic of global platforms, making it necessary to address the economic and ideological dimensions of children's media literacy together. In countries such as Türkiye, which extensively consume global platforms but have limited influence over their production and governance structures, this process acquires particular significance in terms of dependency in digital content production and the homogenization of cultural identity. Consequently, the observations of primary school teachers offer an important lens not only for pedagogical insights but also for understanding the broader impacts of digital culture.

### 2.3 Media literacy in the context of cultural autonomy

Under the dominance of global digital platforms, cultural autonomy has become one of the central objectives of media literacy. Giroux (2020) argues that cultural autonomy is directly related to individuals' capacity to recognize ideologically framed narratives shaped by media and to think critically against them. Children's media experiences are not merely processes of entertainment or learning; they are also sites of identity negotiation, through which children continually redefine who they are, what they value, and how they position themselves in the world (Buckingham, 2008; Livingstone, 2014). For this reason, media literacy may be understood as a pedagogy of resistance that enables children to preserve their own cultural contexts within global media narratives. Kellner and Share (2007) conceptualize media literacy not simply as a skill but as a transformative educational practice that fosters critical awareness against cultural domination. Couldry and Mejias (2019) similarly argue that in the digital age, cultural autonomy must encompass forms of resistance to the colonial structures of data extraction and platform power. In this sense, cultural autonomy does not refer merely to an individual right of choice in media consumption; rather, it denotes the capacity to sustain local values, linguistic diversity, and modes of meaning-making (Appadurai, 1996). When teachers critically engage media content in classroom practices, they mediate the development of such cultural awareness among students (Hobbs, 2011). Thus, media literacy can function as a form of pedagogical resistance against the unidirectional cultural flows imposed by global platforms.

This study is significant in that it examines students' relationships with media and their media literacy skills through teachers' observations. Existing research on children's media experiences has predominantly adopted a risk-reduction and harm-prevention perspective. For example, studies focusing on parental and teacher interventions aimed at protecting children from inappropriate digital content (Nikken and Schols, 2015), as well as studies addressing problematic social media use and social media addiction among children and adolescents (Van den Eijnden et al., 2016), exemplify this approach. Similarly, research demonstrating that media literacy interventions targeting the prevention of cyberbullying enhance children's online safety (Vanderhoven et al., 2014); educational programs aimed at reducing the negative effects of violent games and media on children (Jeong et al., 2012); studies focusing on excessive screen use and digital addiction (Radesky and Christakis, 2016); and media literacy initiatives designed to protect children against online privacy risks and the risks associated with content, contact, conduct, and the understanding of algorithmic and commercial structures (Livingstone et al., 2017; Livingstone and Stoilova, 2021) all represent common examples of this protective framework.

Beyond this dominant perspective, the present study contributes by focusing on how media content (such as news, advertising, and social media) shapes students' value orientations and behavioral patterns, thereby strengthening its theoretical and pedagogical relevance. A second major contribution lies in making visible the multidimensional nature of media literacy from the perspective of teachers. The existing literature on media literacy has largely concentrated on students' skills and performances (Buckingham, 2019a; Livingstone, 2014). By contrast, this study seeks to address this gap by examining whether teachers conceptualize media literacy merely as a technical skill or as a pedagogy linked to cultural autonomy and social resistance.

The fact that the role of global digital platforms in children's lives displays considerable similarity across cultural contexts (Buckingham, 2003; Van Dijck et al., 2018) enhances the international transferability of the study's findings. Discussing the effects of platforms on children within the framework of platform imperialism, based on teachers' perceptions, offers both original and universal contributions to the literature. Examining the cultural and ideological effects of platforms from a teacher-centered perspective provides a critical lens for debates on digital dependency and cultural homogenization.

Finally, the interpretation of media literacy in teachers' views not merely as an individual skill but as a pedagogy of cultural autonomy and social resistance highlights the study's theoretical originality and political contribution. This perspective demonstrates that media literacy in teacher education policies should not be confined to risk-focused issues such as information security, disinformation, or cyberbullying (Livingstone et al., 2017; Radesky and Christakis, 2016), but should also be positioned as a critical domain for fostering ethical values, democratic citizenship, and social responsibility (Giroux, 2014; Kellner and Share, 2019). Recent research also suggests that news literacy interventions at the primary school level can strengthen children's ability to identify misinformation and may support their civic engagement (Polizzi et al., 2026). In this respect, the study contributes to rethinking the content and pedagogical aims of media literacy education and seeks to strengthen teachers' roles as both practitioners and critical thinkers in this process.

The aim of this study is to describe students' relationships with media and their levels of media literacy based on the perspectives of primary school teachers, and to make visible—through teachers' evaluations—the ways in which news, advertising, and social media content are reflected in children's value orientations and behavioral patterns. The study also seeks to interpret the role of global digital platforms in children's everyday lives in relation to debates on platform imperialism, and to examine how teachers conceptualize media literacy within broader debates on cultural autonomy and social resistance. Accordingly, the research questions of the study are as follows:

- How do primary school teachers perceive students' relationships with media and their media literacy skills?
- According to teachers, how are media contents reflected in students' value orientations and behavioral patterns?
- According to teachers, what role do global digital platforms play in children's everyday lives?
- How do teachers position media literacy as a pedagogical response to platform imperialism?

## 3 Method

### 3.1 Research design

This study was designed using a qualitative case study approach. A case study is a research strategy that enables an in-depth examination of a contemporary phenomenon within its real-life context (Yin, 2018). The case examined in this study consists of primary school teachers' observations and evaluations regarding students' relationships with media and their media literacy skills in Türkiye.

A holistic multiple-case study design was adopted (Stake, 2006; Yin, 2018). Each primary school teacher was treated as an individual case observing students' media experiences; however, findings were synthesized across all cases to develop a comprehensive understanding of primary school teachers' perspectives on media literacy. This approach facilitated both an in-depth exploration of individual teacher experiences and the identification of patterns and variations across cases.

The study was conducted within an interpretive paradigm. According to this paradigm, reality is socially constructed, and researchers seek to understand and interpret participants' experiences from their own perspectives (Merriam and Tisdell, 2016). Concepts such as platform imperialism, cultural autonomy, and pedagogical resistance were used as analytical lenses to interpret primary school teachers' narratives, while care was taken not to impose these concepts directly on the participants.

### 3.2 Research setting

This study was conducted in public primary schools affiliated with the Ministry of National Education in Türkiye. The participants were selected from four geographical regions of the country and were employed in schools located in villages, small towns, and city centers. This geographical and residential diversity enabled the inclusion of primary school teachers' experiences from different socio-economic and cultural contexts.

Most of the schools where the participating primary school teachers were employed had basic technological infrastructure. While the majority of schools were equipped with smart boards and internet access, the frequency and purposes of using these technologies varied across schools. However, no specific curriculum or systematic instructional program dedicated to media literacy was implemented; instead, practices related to media literacy education were largely left to primary school teachers' individual initiatives.

The data collection process was carried out through both face-to-face and online interviews, depending on the availability of the teachers and the researchers. Face-to-face interviews were conducted either at the schools where primary school teachers worked or in quiet locations preferred by the participants, while online interviews were conducted via digital platforms. In both formats, priority was given to creating a comfortable and sincere environment in which participants could freely express their views.

### 3.3 Participants

The participants of the study consisted of 71 primary school teachers working at different grade levels. The demographic characteristics of the participants are presented in Table 1. Of the participants, 52 (73.2%) were female and 19 (26.8%) were male. The teachers ranged in age from 25 to 53 years, and their professional experience varied between 1 and 25 years. A substantial proportion of the participants (*n* = 40, 56.3%) had 0–5 years of professional experience, and more than half (*n* = 37, 52.1%) were young teachers aged between 25 and 30.

**Table 1:** Demographic characteristics of the participants.

Variable	Category	Female (*f*)	Male (*f*)	Total (*f*)	Percentage (%)
Professional experience	0–5 years	31	9	40	56.3
	6–10 years	7	4	11	15.5
	11–15 years	4	2	6	8.5
	16–20 years	5	3	8	11.3
Age	21–25 years	5	1	6	8.5
	25–30	28	9	37	52.1
	31–35	13	3	16	22.5
	36–40	3	3	6	8.5
	41–45	5	3	8	11.3
	45 and above	3	1	4	5.6
Gender		52	19	71	100.0

This wide range of experience allowed the inclusion of diverse perspectives on media literacy, encompassing both younger primary school teachers, often considered part of the digital native generation, and more experienced primary school teachers with longer professional trajectories. During the interviews, it was observed that younger primary school teachers demonstrated higher levels of competence in using digital technologies and familiarity with media platforms, whereas more experienced primary school teachers tended to provide deeper reflections in terms of pedagogy and child development.

In the Turkish education system, primary school includes children between the ages of 6 and 10, corresponding to Grades 1–4. Although the participants did not teach older students directly, many worked in school environments where older age groups were also present, allowing broader observations and professional exchanges regarding children's media practices across different age groups. In addition, all participants had taught at least two different grade levels during their professional careers. At the time of the study, there was no legal regulation in Türkiye specifically prohibiting primary school-aged children from using social media platforms such as TikTok or Instagram.

### 3.4 Data collection instrument

Data were collected using a semi-structured interview form. The semi-structured interview technique allows researchers to focus on the research topic through predetermined questions while also providing flexibility for participants to elaborate on their experiences in depth (Kvale and Brinkmann, 2009). The interview form was developed by the researchers in line with the aims and research questions of the study and was finalized after expert review. The interview form consisted of three main sections:

*Demographic information:* Participants' age, gender, professional experience, grade levels taught throughout their professional careers, settlement type, and school characteristics

*Main interview questions:* Students' media use habits, reflections of media content on students' behaviors and values, the role of digital platforms, and the conceptualization of media literacy

*Follow-up questions:* Additional prompts such as “Could you elaborate on this?”, “Can you give an example?”, and “What other observations have you made regarding this issue?” to deepen participants' responses

A pilot implementation of the interview form was conducted with three primary school teachers to test the clarity of the questions. Based on the pilot results, the wording of some questions was refined and additional probing questions were included.

### 3.5 Data collection process

The data collection process was carried out between September 2025 and November 2025. To recruit participants, maximum variation sampling, one of the purposive sampling strategies, was employed (Patton, 2015). This approach aimed to enhance data richness by including teachers from different geographical regions, settlement types (villages, towns, and city centers), and levels of professional experience. Participants meeting these criteria were reached through professional associations, social media groups, and snowball recruitment techniques.

Interviews were conducted either face-to-face or online, depending on participants' availability and location. Face-to-face interviews were conducted with teachers who were geographically accessible to the researchers. These interviews took place either at the schools where the teachers were employed or in quiet settings preferred by the participants. The remaining interviews were conducted online via digital platforms. Each interview lasted approximately 30 min. With participants' consent, some interviews were audio-recorded and later transcribed by the researchers. In cases where participants did not consent to recording, detailed interview and field notes were taken during and immediately after the interview, and these notes were included in the data analysis process. Prior to the interviews, participants were informed about the purpose of the study, the use of data, confidentiality procedures, and the voluntary nature of participation, and written informed consent was obtained.

All interviews were conducted in Turkish. The excerpts presented in the manuscript were translated into English by the researchers. During the translation process, particular attention was paid to preserving the original meaning and contextual integrity of participants' statements.

### 3.6 Data analysis

In case studies, data analysis involves an in-depth description of the case and the identification of cross-case themes (Yin, 2018). In this study, data were analyzed using thematic analysis (Braun and Clarke, 2006), a method used to identify and report themes within qualitative data. The analysis process followed these stages:

*Familiarization with the data:* Audio-recorded interviews were transcribed, and detailed interview and field notes were prepared for non-recorded interviews. The researchers repeatedly reviewed all transcripts and notes to become familiar with the data.

*Generating initial codes:* The data were coded line by line, and meaningful coding units were derived from teachers' statements. The coding and theme development processes were conducted manually by the researchers without the use of qualitative data analysis software.

*Searching for themes:* Codes were clustered to form potential themes, and relationships between codes and themes were examined.

*Reviewing themes:* Themes were reviewed by comparing them with both the coded data and the entire dataset to ensure coherence and consistency. The researchers held regular meetings to discuss and revise themes.

*Defining and naming themes:* The essence of each theme was identified, clear definitions were developed, and appropriate names were assigned.

*Producing the report:* Themes were presented in the findings section and supported by direct quotations.

Both inductive and deductive approaches were used in the analysis process. While themes emerging directly from the data were identified inductively, theoretical concepts such as platform imperialism, cultural autonomy, and pedagogical resistance provided a deductive analytical framework.

### 3.7 Researcher positionality

In qualitative research, it is important to explicitly acknowledge how researchers' experiences, values, and perspectives may influence the research process (Creswell and Poth, 2018). The researchers conducting this study are academics in the field of educational sciences with research experience in literacy, critical pedagogy, and childhood studies. While they hold a critical perspective on the effects of digital platforms on children, they made a deliberate effort to foreground teachers' experiences and perspectives throughout the study.

One of the researchers is also a parent of a child of primary school age and thus has personal experience related to children's media use. While this positionality facilitated a deeper understanding of teachers' observations, it also carried the risk of parental concerns influencing data interpretation. To mitigate this risk, the researchers regularly questioned their own assumptions and engaged in peer debriefing to minimize potential biases.

Although platform imperialism and cultural autonomy were used as analytical lenses, care was taken not to impose these concepts onto teachers' narratives. The theoretical framework served as a tool for organizing and interpreting the data, while participants' own meanings and expressions were prioritized. During analysis meetings, researchers critically evaluated one another's interpretations and discussed alternative explanations.

### 3.8 Quality criteria and ethical principles

In qualitative research, validity and reliability are addressed through the concepts of credibility, transferability, dependability, and confirmability, rather than through quantitative criteria (Lincoln and Guba, 1985). The following strategies were employed to ensure rigor in this study.

*Credibility:* To enhance credibility, prolonged engagement and in-depth data collection were prioritized. Interviews were conducted in environments where participants felt comfortable, and each interview lasted approximately 30 min. The use of a semi-structured interview form enabled participants to express their experiences in detail. In addition, member checking was employed during data collection; unclear or seemingly contradictory statements were returned to participants for clarification. The researchers also met regularly during the analysis process to discuss the consistency of codes and themes and to incorporate multiple perspectives into the analysis.

*Transferability:* To support the transferability of the findings to similar contexts, detailed descriptions (*thick description*) of participants' demographic characteristics, the research context, and the data collection process were provided. The inclusion of teachers from different geographical regions, settlement types (villages, towns, and city centers), and levels of professional experience allows the findings to be considered across diverse contexts. Detailed reporting of the research setting and participants enables readers to assess the applicability of the findings to their own contexts.

*Dependability:* Dependability was ensured through systematic documentation of the data collection and analysis procedures. Regular code comparisons were conducted among researchers, and intercoder agreement was monitored. All stages of the research process—including interview dates, transcription procedures, and analysis steps—were documented in an audit trail.

*Confirmability:* To ensure that the findings reflected participants' experiences rather than researchers' biases, triangulation was employed. Data diversity was achieved by including participants from different regions, levels of experience, and genders. The researchers engaged in continuous reflexivity, critically examining their own assumptions and positionalities throughout the analysis process. Direct quotations were used to ground the findings in participants' own voices.

### 3.9 Ethical considerations

The study was conducted following approval from the Düzce University Scientific Research and Publication Ethics Committee (Approval No. 2025/404). Participants were informed about the purpose of the study, the voluntary nature of participation, confidentiality, and their right to withdraw at any time, and written informed consent was obtained. To protect participants' identities, pseudonyms were used in the findings, and participants were coded as T1, T2, T3, … and T71. Audio recordings and transcripts were stored on password-protected computers and were accessible only to the research team.

## 4 Findings

**Figure 1:**
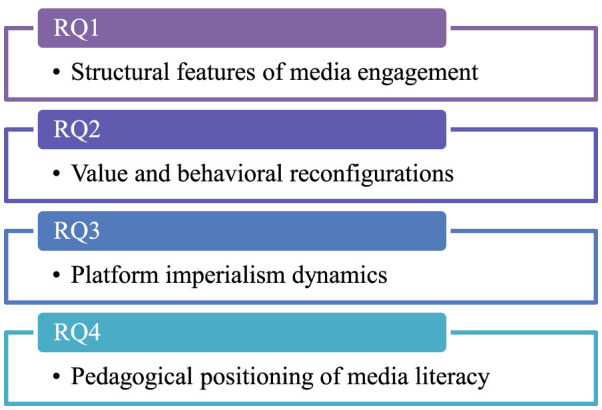
Relational configuration of findings across the four research questions.

To provide an overview of how the findings are structured across the four research questions, Figure 1 presents their relational configuration. Rather than representing isolated thematic blocks, the findings form a layered pattern linking students' media engagement, value transformations, platform-level dynamics, and pedagogical positioning.

### 4.1 Students' media engagement and media literacy

Teachers' observations provide important insights into how primary school students engage with media and how their media literacy skills manifest in everyday educational contexts. In line with the first research question, these observations reveal not only the forms of students' media use but also the qualitative characteristics of this engagement and the limitations they experience in critically and ethically navigating media environments. The themes and categories derived from teachers' accounts are presented in Table 2.

**Table 2:** Thematic patterns in primary students' media engagement and media literacy.

Theme	Category	Description	*f*
Algorithmically structured childhood	Platform-centered concentration	Students' media experiences are concentrated on a limited number of global platforms, with content selection shaped by algorithmic recommendations and trend-driven logics.	38
	Visibility, participation, and socialization	Through content production, seeking likes, and engaging in online interaction, students position themselves as visible and participatory actors within digital environments.	34
	Digitalization of authority	News and information flows increasingly occur through digital platforms rather than through teachers or families, repositioning media as a central source of authority in everyday life.	33
	Normalization of risk	Exposure to violent content, early intensive use, and tendencies toward screen dependency become normalized components of students' media experiences.	11
Fragile critical subjectivity	Lack of critical filtering	Weak verification practices, limited questioning, and insufficient resistance to algorithmically curated content flows.	28
	Erosion of ethical awareness	Limited development of ethical responsibility, neutrality, and value-based reflection in media use and content sharing.	18

The thematic analysis reveals that students' media engagement is not merely a matter of usage patterns but reflects a structurally organized digital environment that reconfigures authority, participation, and critical subjectivity. Teachers' narratives suggest that childhood is increasingly shaped within algorithmically structured media ecosystems, where platform concentration, visibility logics, and digitally mediated authority intersect.

Children's early and intensive engagement with media environments has significantly transformed the school's role as a primary source of knowledge. Access to information is no longer predominantly mediated by teachers but increasingly guided by algorithm-driven flows. As one teacher stated, “Even news flows often come from social media; there are students who see current events before the teacher does” (T10). Similarly, T9 observed that students are “constantly online,” accessing information instantly yet simultaneously being exposed to large amounts of disinformation. This shift represents a critical turning point, compelling teachers to reconsider their pedagogical interventions within a context where epistemic authority is increasingly digitalized.

Teachers further emphasized that students' media experiences are concentrated on a limited number of global platforms. “The current generation is completely immersed in media. Social media, especially TikTok and YouTube, is indispensable in their lives” (T10). These platforms function as dominant entry points, with content selection shaped largely by trending logics and algorithmic recommendations. Such concentration suggests that media engagement is structured less by individual preference and more by platform-centered visibility systems.

Within this environment, students position themselves not only as consumers but also as participatory and visible actors. Practices such as opening personal channels, producing TikTok or gaming videos, and seeking peer approval were frequently reported. As T10 explained, “They are familiar with phones and tablets. They love games… Some of my students even have YouTube channels.” This participatory orientation reflects how recognition, self-presentation, and social validation become embedded within everyday media practices.

At the same time, teachers' observations indicate that qualitative problems in media use cannot be explained solely by individual choices. Rather, digital culture appears to actively reshape children's modes of thinking, attention processes, and perceptions of reality. Teachers described excessive and purposeless engagement: “I observe that they use it excessively, unnecessarily, and without a clear purpose” (T4). T12 noted that students are “weak in understanding whom it serves and how it can manipulate them.” Concerns about insufficient family supervision were also evident: “It is going in a very worrying direction. Unfortunately, families do not control their children” (T7). T2 similarly emphasized that students often use media primarily for entertainment without seeking reliable information.

These patterns suggest that early-age engagement with digital platforms has evolved into a multidimensional structure that directly intersects with cognitive, social, and ethical domains of childhood development. Teachers' accounts of uncontrolled use, lack of critical evaluation, and limited ethical awareness indicate that media engagement extends beyond passive consumption and constitutes a distinct risk domain in the digital era.

In this context, intensive technology use, when not supported by core media literacy components such as cognitive filtering, ethical judgment, and digital autonomy, may systematically undermine learning and developmental opportunities. Teachers' remarks such as “They can believe everything they see on social media” (T41) and that students sometimes believe incorrect information illustrate the fragility of critical filtering processes. Additionally, weaknesses in neutrality, originality, and ethical reflection point to an erosion of value-based awareness in media engagement.

Taken together, these findings demonstrate that media literacy at the primary school level can no longer be considered an “additional skill.” Instead, within algorithmically structured childhood environments, it emerges as an integral component of foundational learning processes. By rendering visible the structural challenges of early media engagement and the pedagogical gaps within school-based media literacy practices, this study contributes to understanding how platform-centered digital ecosystems reshape both childhood experience and educational responsibility.

### 4.2 Media content, values, and behavioral patterns

In line with the second research question, the thematic analysis demonstrates that teachers do not interpret media content merely as producing isolated “reflections” on students' values and behaviors. Rather, their narratives reveal a deeper structural transformation in which media functions as a constitutive cultural logic shaping children's moral frameworks, social norms, consumption orientations, and everyday behavioral repertoires simultaneously. The analysis identified two interrelated patterns that cut across value and behavioral domains: (1) the restructuring of normative frameworks and (2) the reorganization of everyday conduct according to media logics (Table 3).

**Table 3:** Patterns of meaning in the relationship between media content and students' value and behavioral worlds.

Theme	Category	Description	*f*
Restructuring of normative frameworks	Moral ambiguity and normalization	Normalization of what is wrong; legitimization of inappropriate behaviors	39
	Erosion of social norms	Weakening of politeness and respect; increased use of slang; diminished empathy; weakened cultural belonging	35
	Construction of consumption-oriented values	Value production through brands; desire for popular products; pursuit of material status; fame-centered life ideals	27
Reorganization of everyday conduct according to media logic	Internalization of violence and aggression	Normalization of violence; weak anger regulation; peer bullying	36
	Pleasure- and instant-gratification-oriented behavior	Impatience; addictive tendencies; desire for immediate possession	36
	Role modeling and identification dynamics	Imitation of celebrities; aspiration to become an influencer	34
	Transformation in cognitive processes	Attention difficulties; reduced imagination; inability to set goals; concentration problems	26

Teachers' accounts indicate that media content produces significant transformations in students' value systems. In particular, a process of moral ambiguity and normalization emerges. One teacher articulated this concern as follows: “I have students who speak using social media slang. I can say that values are our bleeding wound. They use overly familiar language. Values such as politeness and respect are being lost” (T10). Another teacher stated directly, “Media influences value judgments” (T54). Some teachers explicitly linked screen addiction to moral decline, noting: “As a result of screen addiction, I find children more aggressive and further removed from moral values” (T56).

The erosion of social norms also appeared prominently in teachers' narratives. As one participant observed, “Especially the ‘perfect lives' or trends on social media can negatively affect their self-perceptions and expectations. The use of coarse language can also increase” (T28). Another teacher remarked, “Their conversations during recess change depending on what they watch” (T20), highlighting the direct translation of media content into peer interactions. Similarly, “Everyone learns and experiences everything much faster… There is no such thing as social life among children anymore” (T3) reflects a perceived transformation in forms of social experience.

Teachers further emphasized the early construction of consumption-oriented value frameworks. “Children's only concern is making money and becoming famous…” (T51) captures this shift succinctly. Likewise, “The language of advertising and influencers leads to the early internalization of consumption norms” (T26) and “It changes consumption habits and ways of speaking” (T10) suggest that media discourse penetrates students' value systems. One teacher described this process as “normalized advertising,” explaining: “Students are constantly exposed to advertising not only through social media and videos but also while playing games” (T40). Similarly, statements such as “They want foods containing dozens of harmful ingredients just because they see their favorite characters in advertisements” (T9) and “This year, I observe that they mostly buy items featuring the Stitch character” (T9) concretely illustrate the early internalization of consumer culture.

In terms of behavioral patterns, teachers described how media content reorganizes everyday conduct. “I observe that violent content is reflected in games” (T5), “…negative videos instill harmful behaviors in children” (T3), and “Short video formats trigger impatience and superficial processing. Violence in games and content is reflected in peer interactions” (T19) indicate the internalization of aggression and the normalization of violence.

Pleasure-oriented and addiction-related behaviors were also frequently emphasized. “Short videos and rapid consumption create an impatient generation” (T13), and “Advertisements can also encourage unnecessary spending” (T45) point to a culture of instant gratification. As one teacher noted, “We are talking about media that affects children's eating, drinking, reading—everything” (T9), underscoring the pervasive reach of media content.

Role modeling dynamics were similarly salient. “Today's children learn many behaviors through media and take people they see in media as role models” (T1), “The profession they want to pursue in the future can be content creation on social media platforms” (T16), and “Children imitate the value judgments of the people they watch” (T61) collectively demonstrate the strengthening of identification processes within digital culture.

Finally, teachers reported transformations in cognitive processes. “I think it affects their goals, priorities, manners, and all their perceptions of life” (T5) reflects a broad restructuring of mental frameworks. Similarly, another teacher noted, “Some students become restless very quickly and lose interest when activities do not provide the same rapid stimulation as short-form videos” (T70). At the same time, some teachers acknowledged that positive outcomes are possible with appropriate guidance: “With carefully selected content and guidance, empathy and participation can increase. Positive videos do not produce negative behaviors” (T3).

Overall, teachers position media content not as an external influence but as a structuring cultural logic that simultaneously reshapes normative orientations and everyday conduct. These findings suggest that media operates as a central organizing framework in children's moral, behavioral, and cognitive development, embedding value formation and behavioral regulation within platform-driven cultural dynamics.

### 4.3 Reconfiguring childhood under platform imperialism: structural dominance, algorithmic authority, and tensions in parental mediation

Primary school teachers' accounts of the influence of global digital platforms on children, interpreted within the framework of platform imperialism, reveal three interrelated thematic patterns: the dominance of global digital platforms; the rise of algorithmic authority and information fragility; and the tension in parental mediation between reinforcement and resistance. The themes emerging from these accounts are summarized in Table 4. Rather than representing isolated categories, these themes reflect recurrent patterns of meaning across teachers' narratives concerning how platform power structures children's everyday media experiences.

**Table 4:** Global digital platforms and children's media experiences in the context of platform imperialism.

Theme	Category	Description	*F*
Dominance of global digital platforms	Platform concentration and usage monopolization	Predominant reliance on YouTube, Instagram, Netflix, and TikTok as central platforms in children's media routines	54
	Cultural homogenization and imposition of lifestyles	Following current agendas primarily through platforms, exposure to global content flows, and the marginalization of local contexts in favor of viral and trending content	20
	Consumption-oriented economic steering	Brand following, consumption preferences, and the pursuit of popular products as markers of value and status	19
	Socializing and directive role of platforms	Platforms assuming quasi-parental functions and shaping value transmission and social norms	18
	Digital devices as symbols of power and status	Technological devices functioning as symbolic indicators of prestige and social capital	17
Rise of algorithmic authority and information fragility	Manipulative content and information disorder	Belief in misinformation, lack of verification practices, and exposure to information pollution	29
	Lack of public oversight and institutional authority	Weak or ineffective regulation, absence of sanctions, and limited institutional control over digital platforms	18
Tension in parental mediation: between reinforcement and resistance	Weak parental mediation: Opening space for platforms	Insufficient supervision, lack of content monitoring, and platforms assuming parental roles in value formation	36
	Limited parental media literacy and awareness	Low levels of digital awareness, reliance solely on time restriction.ns, delegating responsibility to schools, and negative role modelling	29
	Protective and empowering parental mediation: producing cultural autonomy	Active guidance, emphasis on cultural values and virtues, and the establishment of boundaries regarding both screen time and content	23

According to teachers, the most widely and dominantly used digital platforms among students are YouTube, Instagram, Netflix, and TikTok. These platforms not only shape children's everyday media consumption but also play a decisive role in influencing how students perceive the world, functioning as globally pervasive digital environments. Teachers emphasized that the intensity of platform use has led to platform concentration and a form of usage monopolization among students.

One of the most significant consequences of this dominance, according to teachers, is cultural homogenization and the imposition of particular lifestyles. Through these platforms, students follow current agendas, are continuously exposed to global content flows, and actively engage with such content. Teachers reported observing that the same lifestyles, habits, and values are implicitly imposed on all users, while local or regional contexts are marginalized in favor of viral and “trending” content—indicating a deliberate form of cultural steering. As one teacher noted, “Students are exposed to the same trends, songs, and expressions online, which creates very similar behavioral patterns among them” (T47).

Teachers further noted that this cultural steering is directly linked to consumption-oriented economic guidance. Students closely follow brands and increasingly perceive ownership of popular products as a marker of status. Another teacher stated, “Children are highly influenced by what becomes popular online and may associate social media visibility with social value” (T44). In this way, platforms are seen as directing economic preferences and normalizing specific consumption patterns. Teachers also emphasized that this process contributes to the transformation of digital devices into symbols of power and status, with technological tools perceived as indicators of social capital among children. Moreover, some teachers underlined that platforms have moved beyond being mere content providers to assume a socializing and directive role that shapes children's value systems and social relationships—sometimes even substituting for parental roles.

Another critical dimension of the influence of global digital platforms, according to teachers, concerns manipulative content and information disorder. Teachers reported that content circulated on these platforms can deliberately disseminate misinformation, leading students to believe inaccurate information and contributing to a pervasive information pollution. Students who lack the awareness or skills to verify and critically assess information tend to accept encountered content as true. This issue was articulated by teachers through statements such as: “They can believe everything they see on social media” (T41) and “They react very quickly, especially to what they see on social media. Sometimes they also believe false information” (T49).

At this point, teachers also drew attention to a lack of public oversight and institutional authority. According to teachers, the absence or ineffectiveness of regulatory mechanisms, sanctions, and institutional monitoring by official bodies facilitates the spread of misinformation and creates conditions in which platforms gain even greater power. This situation was interpreted as a structural weakness that reinforces platform imperialism. Taken together, these accounts suggest a broader pattern in which authority over knowledge and information flows increasingly shifts from traditional institutions toward algorithmically structured digital environments, rendering children's information practices more fragile and less institutionally anchored.

Another prominent theme emphasized by teachers concerns the role of the family in the dominance of global digital platforms over children. Teachers argued that leaving children unprotected in the face of media, the absence of value transmission within the family, and platforms effectively “parenting” children in place of caregivers constitute core factors that strengthen platform imperialism. A lack of parental awareness regarding the content children are exposed to, as well as insufficient guidance, was identified as a major shortcoming.

Teachers reported that many families lack adequate awareness of digital platforms. This was reflected in one teacher's observation: “Families sometimes believe that limiting screen time alone is sufficient, but children also need guidance regarding the content they consume” (T33). Allowing children to use tablets without limits is often perceived as a positive parenting practice, while parents' own excessive engagement with digital media positions them as negative role models. Some teachers also noted that certain families expect schools and teachers to assume responsibility for children's media use, rather than viewing it as part of their own role. These concerns are reflected in statements such as: “Unfortunately, I think adults' excessive media use sets a bad example, and the decreasing amount of family time negatively affects children” (T5), “There are only time restrictions; other than that, no content control is implemented” (T8), “Families' attitudes toward media are decisive for children. Constantly having the television on at home and uncontrolled YouTube use create model behaviors for children. If families are not aware, children's media literacy remains weak” (T14), and “Parents who have difficulty establishing authority at home often expect control from teachers or do not feel the need to control at all” (T6). Additionally, some teachers reported—based on parental accounts—that children may display intense anger reactions and aggressive behaviors when access to digital devices is restricted: “When I speak with families, they say that children sometimes have tantrums, cry intensely, and in some cases even hit, push, or display physical aggression when devices are taken away” (T64). Similarly, another teacher remarked, “When digital devices are taken away, some children react as if a very important part of their daily lives has suddenly disappeared” (T27). At the same time, teachers noted that a small number of families actively attempt to foster critical perspectives by spending time with their children, emphasizing cultural values and virtues, and setting clear boundaries regarding screen time and content.

### 4.4 Pedagogical responses to platform imperialism

In line with the fourth research question, this section examines how teachers position media literacy as a pedagogical response to platform imperialism. As summarized in Table 5, teachers' accounts coalesce around three interrelated pedagogical positionings: Critical Autonomy Against Algorithmic Structuring, Pedagogical Resistance to Platform Power, and Transformative Participation Beyond Platform Logic. The findings suggest that media literacy is framed not as a neutral or purely technical classroom competence, but as a critical educational stance developed in response to platform dominance, algorithmic authority, and digitally mediated power relations. Across these themes, teachers articulate media literacy as a structured pedagogical intervention aimed at cultivating critical verification practices, ethical digital agency, and participatory capacities that extend beyond the behavioral logics embedded within global platforms.

**Table 5:** Pedagogical positionings of media literacy in response to platform imperialism.

Theme	Category	Description	*f*
Critical autonomy against algorithmic structuring	Cultivating verification and skepticism	Encouraging students to question content, verify information, and develop resistance to algorithmically curated information flows	33
	Making algorithmic and ideological logics visible	Explaining how algorithms operate, how targeted advertising functions, and how content is strategically structured	29
	Interpretive analysis of media texts	Analyzing headlines, image–text relations, advertising discourse, and the background logics of trending content	27
Pedagogical resistance to platform power	Countering manipulation and disinformation	Framing media literacy as a defense against misinformation and information disorder	31
	Protecting against harmful and violent digital environments	Raising awareness of cyberbullying, violent media, and unsafe digital spaces	18
	Cultivating ethical and responsible digital agency	Emphasizing ethical media use, awareness of social consequences, and responsible participation in digital environments	14
Transformative participation beyond platform logic	Empowerment through production	Engaging students in project-based production, short films, and alternative content creation	28
	Experiential and participatory learning practices	Drama, scriptwriting, and hands-on pedagogical practices that enable reflective engagement with media	21
	Distributed responsibility across school–family–community	Positioning media literacy as a collective and shared responsibility extending beyond the classroom	17

Teachers frequently described media literacy in terms of fostering critical verification practices and cultivating skepticism toward algorithmically curated content. In their accounts, developing the ability to question information, verify sources, and adopt a reflective stance emerged as a recurring priority. As one teacher explained: “I show them a news image or headline and ask whether they think it is true or false. We need to teach them to ask whether they have verified this news from another source” (T13).

This emphasis on verification was closely linked to teachers' recognition that media environments were not neutral spaces. According to teachers, content was structured through algorithms, advertising strategies, and forms of ideological steering. Consequently, media literacy was positioned not only as the consumption of information but as an effort to make the logic of digital systems visible. As one teacher noted, “We should explain how algorithms work and how advertisements target people” (T18). Across accounts, understanding why particular content appeared before students was presented as an integral dimension of critical autonomy.

Teachers also referred to interpretive engagement with media texts as part of this positioning. News headlines, image–text relationships, advertising language, and the background logics of trending content were described as focal points of classroom inquiry. As one teacher explained, “We discuss why certain headlines are written in attention-grabbing ways and what messages advertisements are actually trying to give” (T52). Through such practices, teachers aimed to move students beyond passive reception toward more reflective engagement with media messages.

A second pattern emerging across teachers' narratives concerned the positioning of media literacy as a form of pedagogical resistance to manipulation and disinformation. Teachers repeatedly referred to students' vulnerability to misinformation and rapid reactions to social media content. The statement “They are unable to understand what other purposes the things they watch or play may serve, or—even if there is no explicit intention behind them—how they may still harm them.” (T15) illustrated how teachers framed the need for systematic questioning and source evaluation. Within this pattern, resisting misinformation was not described merely as an individual skill, but as a responsibility connected to students' participation in broader information environments.

Teachers also positioned media literacy as protective in relation to harmful and violent content. Raising awareness of cyberbullying, violent media materials, and inappropriate digital environments was described as part of classroom practice. As one teacher noted, “We try to explain that cyberbullying can hurt people just as much as face-to-face bullying” (T39). These accounts suggested that media literacy was understood as offering cognitive, ethical, and psychosocial forms of protection within platform-dominated contexts.

Another recurring emphasis concerned ethical and responsible digital agency. Teachers stressed that students should not engage with media content solely as consumers, but as individuals capable of considering its social consequences. One teacher emphasized, “Students should learn that sharing something online can also have consequences for other people” (T70). Ethical media use was addressed through classroom discussions of how media content affected individuals and communities, reinforcing the connection between digital participation and social responsibility.

The third pattern reflected teachers' positioning of media literacy as enabling forms of participation that extended beyond the behavioral logics embedded within global platforms. Teachers described media production practices—such as project development, short film production, and content creation—as opportunities for students to move from passive consumption toward active engagement. These activities were framed as pedagogical practices that supported students' ability to express perspectives and construct meaning.

Teachers further associated experiential and participatory learning approaches with this transformative dimension. Through drama activities, scriptwriting, and hands-on classroom practices, students were encouraged to experience, reflect on, and reinterpret media messages.

Finally, several teachers emphasized that media literacy could not be confined to classroom instruction alone. The role of families and broader social contexts was frequently mentioned. As one teacher stated: “School is already the place where they cannot access these tools; the real responsibility lies with the family. The difference is clearly visible. There are no problems among children from conscious families” (T29). This account reflected a pattern in which media literacy was positioned as a distributed responsibility across school, family, and community contexts.

Overall, the findings presented in Table 5 indicated that teachers positioned media literacy not as a narrowly defined technical competence, but as a pedagogical orientation shaped by concerns about algorithmic structuring, platform power, and students' participation in digital environments. Across narratives, media literacy emerged as a response developed within the everyday realities of platformized childhood, emphasizing verification, ethical awareness, and participatory engagement.

## 5 Conclusion and discussion

This study set out to examine primary school teachers' observations of students' media engagement, the ways media content is reflected in children's value orientations and behavioral patterns, the role of global digital platforms in everyday childhood experiences, and how media literacy is positioned pedagogically in response to platform imperialism. Rather than treating these dimensions as isolated domains, the findings reveal interrelated patterns of meaning that point to a broader reconfiguration of childhood under platform-dominated digital conditions. When read together, the four research questions converge around a central structural issue: the algorithmic organization of media environments and its implications for authority, value formation, and pedagogical responsibility.

Policy documents, as platforms through which global media companies declare their commitment to principles such as community and freedom of expression, may appear to promote value discourses centered around themes including expression, community, safety, choice, and development. Nevertheless, by selectively assigning responsibility for the implementation of these values and frequently shifting this burden onto users, platforms limit their own responsibility in putting these principles into practice (Scharlach et al., 2023). This situation renders child users of digital platforms particularly vulnerable to the risks identified by Livingstone and Stoilova (2021).

Although the effects of global digital platforms on children are widely discussed in the literature, these debates are often conducted at a macro level. The present study contributes by rendering visible how such transformations materialize in everyday primary school contexts through teachers' pedagogical experiences. Teachers' accounts consistently indicated that children's media engagement is increasingly organized within platform-centered and algorithmically curated environments. In this sense, childhood appeared less as a sphere of autonomous exploration and more as a space structured by visibility logics, trending mechanisms, and recommendation systems.

This pattern resonates with the broader literature on the “platform society” (Van Dijck et al., 2018), which conceptualizes digital platforms as infrastructural environments shaping economic, social, and civic processes. Teachers' observations that students encounter news and information first through social media rather than through teachers or families illustrate how epistemic authority is increasingly digitalized. This shift does not merely represent a change in information channels; it signals a transformation in the structure of authority itself. Traditional institutional mediators—such as schools, families, and official sources—are increasingly losing their centrality in shaping and regulating knowledge flows. Instead, algorithmically structured flows reorganize how children access, interpret, and prioritize information. As Head et al. (2020) also emphasize, in an era in which algorithms permeate everyday life, students need to develop a more comprehensive understanding of how news and information circulate, how they are shaped by algorithm-driven intermediaries operating through market logics, and how they function within society.

The findings therefore suggest that the issue is not simply children's “excessive media use,” but the structural relocation of authority within digitally mediated environments. In this respect, the study aligns with Jin (2015) conceptualization of platform imperialism, which highlights the reproduction of unequal power relations within global digital economies. In the Turkish context, where global platforms are extensively consumed yet not domestically governed, teachers' narratives reflect a local manifestation of these structural asymmetries.

The study's findings further demonstrate that children's media engagement is closely linked to processes of homogenization at both cultural and behavioral levels. Consistent with Kaya and Kiliç (2024) argument regarding the convergence and homogenization of platform features, teachers described children's concentration on a limited set of global platforms and the circulation of similar content, values, and aspirations across contexts. Homogenization appeared not only as similarity of content but also as convergence in behavioral repertoires, consumption patterns, and forms of self-presentation.

Raffoul et al. (2023) show that social media platforms generate billions of dollars annually from youth audiences, underscoring children's economic value within platform capitalism. Teachers' observations regarding brand-centered aspirations, influencer role models, and fame-oriented life ideals reflect how this economic logic penetrates children's value worlds. In this sense, children are positioned not merely as users but as both consumers and potential producers of value within platform economies—a dynamic that resonates with Holloway (2019) analysis of children within surveillance capitalism.

This economic positioning is not limited to consumption; in some cases, children also become active participants in platform economies through content production. In particular, the production of “play”-themed videos on platforms such as YouTube illustrates how the boundary between play and labor becomes increasingly blurred in digitally mediated environments. Freitas et al. (2024) argue that although such digital content production may appear as spontaneous play, it is often embedded within value-generating platform logics and raises significant ethical concerns regarding children's rights. Their analysis demonstrates that when children's visibility, creativity, and affective engagement are monetized within platform infrastructures, the distinction between leisure and economic activity becomes less clear. These dynamics also reflect broader forms of economic extraction within platformized environments, where children's attention, participation, and visibility may become integrated into data-driven systems of value production. In this respect, teachers' observations regarding students' aspirations to become influencers and engage in content creation can be interpreted as classroom-level reflections of a broader structural transformation in which children are positioned not only as consumers but also as potential producers of economic and symbolic value within global platform ecosystems.

Moreover, the restructuring of normative frameworks observed in teachers' narratives suggests that media functions as a cultural pedagogy (Giroux, 2020). The normalization of violence, erosion of social norms, and construction of consumption-oriented value systems indicate that media content operates not as a peripheral influence but as a structuring cultural logic shaping moral and social orientations. This interpretation aligns with Buckingham (2008, 2019b) argument that children's identity formation and cultural belonging are increasingly mediated within digital environments shaped by global platform logics.

Importantly, teachers' accounts did not reduce these transformations to individual moral failings. Rather, they pointed to a patterned restructuring of everyday conduct in alignment with media logics—instant gratification, visibility seeking, algorithmic popularity, and symbolic status attached to digital devices. This reinforces the argument that homogenization is both cultural and structural, embedded within platform architectures and economic incentives rather than merely within children's preferences.

A second major pattern emerging across findings concerns information fragility. Teachers repeatedly referred to misinformation, lack of verification, and students' rapid acceptance of content encountered on social media. Livingstone et al. (2017) emphasize that children's developmental capacities may limit their ability to critically distinguish between content types in digital environments. The present findings extend this argument by situating such vulnerability within algorithmically structured attention economies.

Teachers' references to weak regulation and insufficient institutional oversight echo Van Dijck et al. (2018) claim that platforms function as public infrastructures without being fully subject to democratic accountability. In this context, misinformation cannot be reduced to an individual cognitive deficit. Rather, it appears as a systemic outcome of platform architectures that blend news, advertising, entertainment, and ideological content within the same continuous flow.

Accordingly, the study suggests that disinformation should be conceptualized as a structural issue intertwined with algorithmic steering, platform logics, and regulatory gaps. The perceived vacuum of institutional authority described by teachers represents not merely policy insufficiency but a reconfiguration of power in the digital information ecosystem This interpretation is also consistent with recent evidence showing that recommendation systems can shape young people's digital consumption practices and intensify exposure to harmful content patterns, thereby increasing the salience of algorithmically structured vulnerability (Regehr et al., 2025).

The findings also reveal that family mediation operates as a site of tension rather than uniform influence. Teachers' narratives indicated that weak parental mediation—limited supervision, reliance solely on time restrictions, and delegation of responsibility to schools—tended to open space for platforms to assume quasi-parental roles in value transmission. This observation resonates with Livingstone and Blum-Ross (2020) analysis of how parental hopes and fears shape children's digital experiences, often unevenly.

At the same time, teachers described instances of protective and empowering mediation practices in which families actively guided content choices, emphasized cultural values, and set boundaries regarding both duration and substance of media engagement. This duality suggests that parental mediation may either reinforce platform dominance or generate partial forms of cultural resistance. Such tension underscores that platform imperialism is not experienced uniformly; rather, it is negotiated within household contexts shaped by varying levels of awareness and agency. This interpretation is consistent with parental mediation theory discussed in the literature. According to Clark (2011), this theory assumes that interpersonal interactions established between parents and children around media function as a central mechanism in children's socialization processes. Accordingly, although the theory originally emerged from scholarly concerns regarding the harmful effects of media, it also sought to explore how constructive actors within children's immediate environments—particularly parents and their intentional mediation practices—might balance or buffer the presumed negative effects of television on children's cognitive development. This point is compatible with recent findings suggesting that parental media literacy may support children's learning-related outcomes through more reflective and democratic parenting practices, indicating that family mediation can also function as a protective condition (Zhou et al., 2025).

The fourth research question addressed how teachers positioned media literacy in response to these structural transformations. The findings indicate that teachers did not conceptualize media literacy as a narrow technical competence. Instead, they framed it as a pedagogical response to algorithmic structuring, misinformation, and platform-centered authority.

This positioning aligns with Kellner and Share (2007, 2019) understanding of critical media literacy as a practice that renders economic and ideological structures visible. Teachers' emphasis on explaining how algorithms operate and how advertising targets users reflects an effort to make platform logics explicit within classroom settings. In this sense, media literacy functions as an intervention aimed at cultivating critical autonomy rather than simply managing screen time. This finding supports the argument advanced by Garcia et al. (2013), who contend that critical media literacy should move beyond traditional approaches that conceptualize media literacy primarily as a process of consumption and instead develop toward a more production-oriented pedagogy.

Similarly, the framing of media literacy as resistance to manipulation resonates with Hobbs's (2017) argument that media literacy plays a central role in combating misinformation in democratic societies. Teachers' focus on ethical responsibility and participatory production further reflects Freire's (1970) view that critical consciousness develops through active engagement rather than passive reception.

However, in line with the reviewer's concern about thematic clarity, the study does not claim that teachers uniformly enacted a coherent critical pedagogy. Rather, the findings reveal recurring patterns in how teachers articulated media literacy as a structured response to platformized environments. These patterns suggest an emerging pedagogical orientation shaped by lived classroom realities rather than solely by abstract theoretical commitments.

Taken together, the findings indicate that media literacy at the primary school level cannot be addressed solely through risk-prevention frameworks focused on addiction, cyberbullying, or screen time management (Nikken and Schols, 2015; Radesky and Christakis, 2016; Vanderhoven et al., 2014). While such concerns remain relevant, teachers' narratives demonstrate that the more fundamental issue concerns the structural embedding of childhood within platform-centered ecosystems.

**Figure 2:**
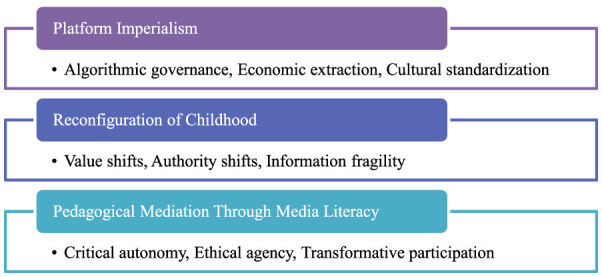
Multi-level synthesis of platform imperialism, childhood reconfiguration, and pedagogical mediation.

The study therefore suggests that media literacy education should incorporate critical engagement with algorithmic steering, economic logics of platforms, ethical responsibility, and the reconfiguration of institutional authority. In teacher education programs, this implies moving beyond purely technical digital skills toward broader questions of cultural autonomy and power relations, a perspective consistent with the critical media literacy literature (Giroux, 2014; Kellner and Share, 2019). To synthesize these multi-level dynamics, Figure 2 presents a conceptual overview of how platform imperialism operates structurally, reconfigures childhood experiences, and elicits pedagogical mediation through media literacy.

Figure 2 brings together the study's findings by illustrating the interconnections between structural platform dynamics, transformations in children's everyday media experiences, and teachers' pedagogical positioning of media literacy. At the structural level, platform imperialism operates through algorithmic authority, economic extraction logics, and the digitalization of information flows. At the level of childhood experience, these dynamics manifest as homogenization of values, normalization of risk, shifts in epistemic authority, and reconfigured patterns of participation and visibility. At the pedagogical level, teachers respond by fostering critical verification practices, ethical awareness, resistance to manipulation, and participatory engagement. The figure does not depict a linear causal sequence; rather, it visualizes a layered and relational configuration in which structural conditions, lived experiences, and educational mediation are mutually implicated.

Taken together, the findings indicate that platform imperialism is experienced in primary school contexts not merely as a technological condition but as a restructuring of authority, value formation, and everyday conduct within digitally mediated environments. Teachers' narratives reveal that media literacy emerges as a pedagogical space in which these structural tensions can be rendered visible, interrogated, and partially negotiated. Rather than positioning media literacy as a comprehensive solution to platform power, the study frames it as a necessary, though not sufficient, domain for supporting children's critical engagement within algorithmically organized environments. By grounding debates on platform imperialism in teachers' lived pedagogical experiences, the study offers an empirically situated contribution to discussions of childhood, digital culture, and education, and underscores the need to understand media literacy as a structurally informed educational response rather than a purely individual skill set.

### 5.1 Limitations

This study examines primary school students' relationships with media and media literacy through teachers' perspectives. Accordingly, the data reflect teachers' observations and pedagogical evaluations of students' media experiences rather than the direct experiences of students or their families.

Interviews were based on teachers' personal experiences and perceptions; therefore, the findings may have been shaped by teachers' individual pedagogical approaches, professional backgrounds, and personal media use. However, this subjective positioning also constitutes one of the core elements of the study, as its primary focus is on teacher perceptions.

Finally, the study was conducted within a specific time frame. Given the rapidly evolving nature of digital platforms, teachers' views represent a particular temporal snapshot, which limits the ability to capture the long-term effects of transformations in the digital media landscape.

## Data Availability

The raw data supporting the conclusions of this article will be made available by the authors, without undue reservation.
